# Management of allergic rhinitis improves clinical outcomes of difficult-to-treat tic disorders or attention-deficit/hyperactivity disorders 

**DOI:** 10.5414/ALX400588E

**Published:** 2023-10-23

**Authors:** Rui-Li Yu, Jing Wang, Xue-Song Wang, Hong-Tian Wang, Xue-Yan Wang

**Affiliations:** 1 Department of Allergy, Beijing Shijitan Hospital, Capital Medical University,; 2 Department of Pediatrics, First Medical Center, PLA General Hospital, Beijing, and; 3 Department of Otolaryngology, Binzhou Central Hospital Affiliated to Binzhou Medical College, Binzhou, Shandong, China

**Keywords:** allergic rhinitis, tic disorder, attention- deficit/hyperactivity disorder (ADHD), Yale Comprehensive Tic Severity Scale (YGTSS)

## Abstract

Aims: This case series aimed to evaluate the effects of treatment for allergic rhinitis (AR) in AR-diagnosed children with previous diagnosis of tic disorders/attention-deficit/hyperactivity disorders (TD/ADHD) but unresponsive to behavioral or medical treatment. Materials and methods: Between July 2016 and June 2021, children diagnosed with AR in our hospital were enrolled. All were diagnosed with TD/ADHD refractory to behavioral or medical treatment. The demography and clinical information were collected from medical records. The outcomes were visual analogue scale (VAS) for AR severity, Yale Comprehensive Tic Severity Scale (YGTSS) for TD symptoms, and Attention-Deficit Hyperactivity Screening Scale (SNAP-IV) for ADHD symptoms. Results: A total of 27 children (18 boys, 9 girls) were included, with a mean age 7.4 ± 2.9 years (3 – 17 years). They had undergone behavioral or medical treatment of TD/ADHD for 3.6 ± 1.9 years but without significant improvement in TD/ADHD symptoms. After 2-6 months of systematic treatment for AR, VAS was decreased to 0.4 ± 0.1 from 0.8 ± 0.2, YGTSS to 3.5 ± 0.7 from 6.8 ± 1.4, and SNAP-IV to 0.4 ± 0.1 from 0.6 ± 0.2 (all p < 0.001). No recurrence of TD/ADHD symptoms was reported during a mean follow-up of 2.4 ± 1.1 years (0.5 – 5 years). Conclusion: AR treatment improves TD/ADHD outcomes in children with difficult-to-treat TD/ADHD. In TD/ADHD children who are unresponsive to behavioral or drug treatment and have AR-related symptoms, AR examination and treatment are recommended for better prognosis.

## Introduction 

Allergic rhinitis (AR) is a common allergic disease in children. AR seriously affects their quality of life, learning, leisure time, and school attendance. Many studies have shown associations between allergic diseases and tic disorder/attention-deficient/hyperactivity disorders (TD/ADHD). Children with TD or ADHD have a high prevalence of allergic diseases including AR, asthma, atopic dermatitis, and allergic conjunctivitis [1, 2, 3, 4, 5, 6, 7, 8, 9, 10, 11]. Children with atopic diseases also have a higher chance of developing ADHD [3, 6, 8], and the severity of ADHD symptoms is correlated with severity and duration of AR [3, 10]. 

TD is characterized by locally occurring, involuntary, frequent, stereotyped muscle twitch. Motor tics usually occur with rapid muscle contraction movements of the fingers, face, neck, shoulders, trunk, and extremities; vocal tics usually occur with contractions of the mouth, nose, throat, and respiratory muscles, and vocalization via airflow through the nose, mouth, and throat. The prevalence of TD in China is ~ 4 – 23% [12]. It is more common in preschool and young school children. The onset of TD most often occurs at the age of 4 – 8 years, with an average age of 6 years old, and the disease severity is highest in 10 – 12 year olds. ADHD is mainly characterized by three core symptoms, including attention deficit, hyperactivity, and impulsivity. The prevalence rate of ADHD among children in China is 6.26% [13], but the consultation rate is only 10%. ADHD usually starts before the age of 6, affecting children’s cognition, emotion, behavior, quality of life, and social function. 

Previous studies reported that the ADHD scores of drug-naïve ADHD children with AR symptoms were improved after AR treatment [10, 11]. This case series included TD/ADHD children diagnosed with AR who were unresponsive to behavioral or medical treatment for TD/ADHD. Their TD/ADHD symptoms were significantly improved after systemic treatment for AR in an otolaryngology department. 

## Materials and methods 

### Patients 

Between July 2016 and June 2021, children diagnosed with AR with a previous diagnosis of TD/ADHD were enrolled in this case series. Inclusion criteria: 1) previous diagnosis of TD/ADHD in the psychiatry specialist hospital or children’s hospital. The diagnostic criteria for TD/ADHD by pediatric psychiatrists were clinically assessed in accordance with the American Mental Illnesses Diagnostic and Statistical Manual 5^th^ Edition (DSM-5) or Chinese Mental Disorders and Diagnostic Criteria 3rd Edition (CCMD-3) [14, 15, 16, 17]. 2) AR diagnosis according to the Guidelines for the Diagnosis and Treatment of Allergic Rhinitis [11, 18]. The results of a skin prick test should be at least 1 allergen ≥ ++ and/or serum sIgE ≥ grade 2. 3) AR treatment in our department. Patients’ symptoms of AR and TD/ADHD were evaluated before and after AR treatment. The study was reviewed and approved by the local ethics committee. All parents signed a written informed consent form. 

### TD/ADHD symptom scores 

The level of TD/ADHD symptoms was evaluated with relevant quantitative data included total Yale Comprehensive Tic Severity Scale (YGTSS) score and Attention-Deficit Hyperactivity Screening Scale (SNAP-IV) scores. The YGTSS score used for the assessment of TD was based on a total score of 100, with 1 – 25 rated as mild, 25 – 50 as moderate, and > 50 as severe [19]. The SNAP-IV score was a 26-item questionnaire completed by parents and teachers as an effective tool for assessing ADHD-related symptoms, where a score of 0 – 1 is normal, 1.1 – 1.5 is borderline, 1.6 – 2 is moderate, and > 2 is severe [20]. 

### AR symptom score 

The evaluation of AR severity was based on the visual analogue scale (VAS) score data of nasal symptoms before and after treatment. VAS < 5 cm (2.4 – 5.0) is mild rhinitis, > 6 cm (5.3 – 7.7) is moderate to severe rhinitis [21]. 

### Statistical analysis 

For basic characteristics of subjects, continuous variables are presented as means with standard deviations (SDs); categorical variables are presented as counts and percentages. Paired t-test was used to compare the AR symptom scores and TD/ADHD scores. Two-tailed p < 0.05 indicates statistical significance. The statistical analyses were performed with SAS software version 9.2 (SAS Institute Inc., Cary, NC, USA). 

## Results 

A total of 27 children (18 boys and 9 girls) were included. The demographic and clinical characteristics at admission to the otolaryngology outpatient department are listed in Table 1. The average age was 7.47 ± 2.9 years (age 3 – 17 years). Six cases denied family history, and 21 cases had a clear family history of allergic diseases: In 5 the father had an allergy history, in 9 the mother, and in 7 both. The main symptoms were stuffy nose in 14 patients; itchy nose and sneezing in 14 cases. All 27 children were strongly positive for more than two perennial allergens using skin prick test or serum total IgE and sIgE detection, including mites (92.6%), food (70.4%), pollen in spring (70.4%) and autumn (77.8%). All 27 patients had moderate-to-severe perennial AR symptoms with seasonal aggravation. Antiallergics were administered in all cases, while additional sublingual desensitization was performed in 4 cases, subcutaneous immunotherapy in 13 cases, and subcutaneous injection of omalizumab in 15 cases [22]. The AR symptoms were improved after systematic and standardized treatment for 3.5 ± 1.6 months (2 – 6 months). VAS scores were significantly decreased to 0.4 ± 0.1 from 0.8 ± 0.2 (p < 0.001) (Table 2). No recurrence was reported during a mean follow-up of 2.4 ± 1.1 years (0.5 – 5 years). 

All patients were previously diagnosed with TD/ADHD in a psychiatric clinic, including 19 with TD, 6 with TD/ADHD, and 2 with ADHD. All had undergone behavioral or medical therapy for TD/ADHD for 3.6 ± 1.9 years (0.5 – 9 years) but without further improvement. After AR treatment, YGTSS (from 6.8 ± 1.4 to 3.5 ± 0.7, p < 0.001) and SNAP-IV (from 0.6 ± 0.2 to 0.4 ± 0.1, p < 0.001) scores were significantly improved (Table 2). 

## Case presentation 

### Case 1 

The patient was a 13-year-old boy who had frequently rubbed his nose, blinked, opened his mouth, and coughed for 8 years. Seven years ago, he was diagnosed with chronic TD at the local pediatric psychiatry department, but the psycho-behavioral therapy did not achieve satisfactory effects. His TD symptoms worsened, manifested as motor tics and vocal tics, with normal electroencephalogram and cranial magnetic resonance, normal intelligence tests, and a YGTSS score of 67. He was diagnosed with Tourette syndrome. Two tablets of Changma Xifeng 3 times a day were administered, but no effect was obvious after 1 year of treatment. The medication was changed to tiapride hydrochloride tablets 50 mg, 3 times a day, but there was still no improvement of symptoms after half a year. He visited the otolaryngology outpatient clinic 5 years ago. Supplementary medical history after inquiries showed that he had recurrent eczema as a baby which was significantly improved after the age of 1, but urticaria often occurred. His father was diagnosed with TD in childhood, and was allergic to dust, cold air, and crabs. His younger sister had eczema, frequent nose itching, sneezing, and nasal congestion. The patient’s current symptoms were nasal itching, sneezing, runny nose, and nasal congestion. The nasal mucosa was pale and edematous, the inferior turbinate was hypertrophied, and the conjunctiva was slightly congested. Skin prick test showed house dust mite +++, dust mite ++++ (“Dust mite” includes both indoor and outdoor dust mite, while “house dust mite” refers specifically to indoor), elm +, birch +, mugwort +++, ragweed +, shrimp ++, crab ++. Peripheral blood eosinophils were 8.3% (reference value 1 – 5%, 0.05 – 0.3 ×10^9^/L). Serum total IgE 79 IU/mL (reference value < 100 IU/mL), sIgE showed house dust mite grade 4, dust mite grade 5, German cockroach grade 1, mugwort grade 3, ragweed grade 1, shrimp grade 2, crab grade 1. Preliminary diagnosis was seasonal aggravation of perennial AR. 

The treatments were: 1) Control of home environment, reduce dust mites, monitor and remove mites at home once every 2 – 4 weeks; wear masks and anti-pollen glasses when going out in spring and autumn; 2) mometasone furoate nasal spray, once daily; montelukast sodium chewable tablets 5 mg and cetirizine hydrochloride drops 5 mg, orally, once a night; 3) after allergy season or after allergy symptoms were controlled, sensitivity treatment was stopped; 4) nasal wash with normal saline 2 – 3 times/day; 5) tiapride hydrochloride, 50 mg, 3 times a day. After using those treatments for 1 month, the patient’s AR symptoms were significantly improved by 70%. Then, the dose of tiapride hydrochloride tablets was reduced from 50 mg twice a day for a week to 50 mg once a day for a week, and then discontinued. After treatment for 3 months, neither AR symptoms nor blinking, mouth opening, throat clearing, or other tics were observed. After treatment for 6 months, antiallergic drugs were only used when the symptoms were aggravated by cold air, and only sublingual desensitization treatment with dust mite drops was used. Sublingual desensitization therapy has been performed for 5 years, and both AR and TD/ADHD symptoms are well controlled. 

### Case 2 

This 11-year-old girl had limb hyperactivity, winking, nose rubbing, puffed cheeks, inattention, and poor academic performance for 9 years. The child has winked and rubbed her nose frequently since she was 2 years old. It was obvious in spring and autumn, and it gradually became more and more perennial. At 6 years old, she repeatedly puffed out her cheeks. At 7 years old, she often coughed. At 8 – 9 years old, she repeatedly shook her head and stretched her limbs. When she was 4 years old, she was diagnosed with Tourette syndrome combined with mixed ADHD. The EEG, dynamic EEG, cranial magnetic resonance, and intelligence test were normal. The YGTSS was 73 points, SNAP-IV was 68 points. Psycho-behavioral therapy has been carried out until the age of 6, but no effect was obvious. After the age of 6, she used clonidine transdermal patch 1 mg/once a week and Ning-dong granules 6 g orally twice a day for half a year. Due to the lack of obvious effect, haloperidol was used from the initial dose of 0.5 mg to 1 mg orally twice a day for 1 year, but the symptoms only improved by 30 – 40%. At the age of 8, she changed to aripiprazole tablets 10 mg/day for 1 year. The frequency of limb hyperactivity decreased to about once every 10 – 20 minutes, but the symptom improvement remained at 30 – 40%. The patient’s academic performance declined year by year, and she came to our hospital for further diagnosis and treatment. She had a past history of eczema, urticaria, and contact dermatitis. Both mother and younger brother had a history of AR. Supplementary medical history after questioning found that the child suffered from shortness of breath, wheezing, frequent coughing and grunting, eyelid and facial twitching, shrugging, long-term night-time snoring, mouth breathing, often waking up, often sleeping on the stomach, often ignoring questions from parents and teachers, and a significant drop in academic performance. Physical examination showed red and swollen nasal mucosa, hypertrophy of inferior turbinate, obvious shadow of the lower eyelids, and large grade 3 tonsils. Skin prick test showed house dust mites and dust mites ++++, *Alternaria* ++, juniper +++, willow ++, sycamore, ashwagandha +, *Artemisia maxima* ++++, *Humulus japonicus* +++, egg white, peanuts, milk +. Serum total IgE 760 KAU/L, sIgE house dust mite grade 5, dust mites grade 6, mold combination grade 6, mugwort grade 5, ragweed grade 4, eggs, peanuts, and milk grade 1. FeNO 39 ppb, pulmonary function test results showed mild obstructive ventilatory dysfunction, FEV_1_ improvement rate was 13.6%, bronchodilation test was positive. Video nasolaryngoscopy revealed hypertrophic adenoids occupying 4/5 of the posterior nostrils, and the airway was significantly narrowed. Pure tone audiometry showed bilateral conduction deafness, air conduction-bone conduction examination was 20 dBHL, and acoustic immittance test binaural B-type chart. Preliminary diagnosis: allergic rhinitis, allergic asthma, adenoid hypertrophy, tonsil hypertrophy, childhood snoring, bilateral secretory otitis media. 

The treatments were: 1) Control of home environment to reduce dust mites and molds, measure and remove mites at home once every 2 – 4 weeks, wear masks and anti-pollen glasses when going out in spring and autumn; 2) mometasone furoate nasal spray, oral montelukast sodium chewable tablets, cetirizine hydrochloride drops, eucalyptus lime and pinene soft capsules; budesonide formoterol powder inhalation 4.5 μg, 2 times a day. 3) after the lung function was significantly improved, subcutaneous injection of omalizumab 300 mg was administrated once a month; 4) adenoidectomy, tonsillectomy, and bilateral middle ear cannulation under general anesthesia; 5) sublingual desensitization treatment with *Dermatophagoides farinae* drops; 6) nasal wash with normal saline 2 – 3 times a day; 7) aripiprazole tablets 10 mg/day were continued, and the dose was gradually reduced after the condition was stable. After 4 omalizumab administrations, the tic symptoms and hyperactivity disappeared; after 14 omalizumab administrations, all symptoms were in good control, and her academic performance was significantly improved. 

## Discussion 

Studies have ﻿shown that ADHD scores are significantly improved in drug-naïve ADHD children with AR symptoms after treatment of AR [11]. The present study further showed that AR treatment significantly improves outcomes in children with refractory TD/ADHD diagnosed with AR. Many studies have noticed that AR is correlated with TD/ADHD [2, 3, 5, 6, 10, 11]. The prevalence of AR in TD/ADHD children is high and vice versa. However, it is not clear whether the relationship between AR and TD/ADHD is a causality or comorbidity. The common features of AR and TD/ADHD include a gradually increasing prevalence, genetic predisposition, environmental factors, similar clinical manifestations, and neuropsychological problems [3, 12, 13, 23]. When ADHD is accompanied by AR, the variety and complexity of ADHD symptoms may increase. 

In the present study, skin prick test showed that mite is the main allergen (92%), and desensitization therapy efficiently improved tic symptoms and hyperactivity in 81.5% of patients after cleaning environment. Yang et al. [10] reported that allergic disease with allergic sensitization is associated with ADHD, and the main allergen is house dust mite. Taken together, mite sensitization is likely to be correlated with the developing TD/ADHD symptoms, therefore AR treatment improves TD/ADHD scores in children who are unresponsive to behavioral and medical treatment of TD/ADHD. Further study is needed for clarification. 

The study has several limitations. First, it has an inherent limitation due to its retrospective nature. Second, the sample size was small. Third, there was no placebo group. Although the reasons why the included TD/ADHD children were unresponsive to behavioral or medical treatment remains further investigation, their TD/ADHD scores were significantly improved after systemic AR treatment. 

## Conclusion 

AR treatment improves clinical outcomes of difficult-to-treated TD/ADHD in children with AR symptoms. In TD/ADHD children who are unresponsive to behavioral or drug treatment for TD/ADHD and who have AR-related symptoms, a family history of allergic diseases, or an aggravation of seasonal symptoms, AR examination and treatment are recommended for better prognosis. 

## Research involving human participants and/or animals 

The study was reviewed and approved by the ethics committee of Beijing Shijitan Hospital, Capital Medical University. 

## Informed consent 

All parents gave their written informed consent. 

## Authors’ contributions 

Rui-Li Yu: literature research; clinical studies; experimental studies; data acquisition; data analysis; statistical analysis; manuscript preparation. 

Jing Wang: definition of intellectual content; clinical studies; experimental studies; statistical analysis; manuscript editing. 

Xue-Song Wang: literature research; experimental studies; data acquisition; data analysis; statistical analysis; manuscript editing. 

Hong-Tian Wang: guarantor of integrity of the entire study; study concepts; study design; definition of intellectual content; manuscript review. 

Xue-Yan Wang: guarantor of integrity of the entire study; manuscript editing; manuscript review . 

All authors read and approved the final manuscript. 

## Funding 

This study was supported by the Project of Beijing Key Laboratory for clinical rational drug use biometric spectroscopy evaluation (2020-KF08); Joint key project of Beijing Municipal Education Commission and Beijing Municipal Natural Science Foundation (KZ202110025030). 

## Conflict of interest 

All authors declare to have no conflict of interest. 

**Table 1.** Demographic and clinical characteristics.

**Variable**	**Mean ± SD (min – max) or N (%)**
Gender	
Boy	18 (66.7)
Girl	9 (33.3)
Age	7.4 ± 2.9 (3 – 17)
Family history of allergy	
No	6 (22.2)
Father	5 (18.5)
Mother	9 (33.3)
Parents	7 (25.9)
Asthma	
Yes	15 (55.6)
No	12 (44.4)
Main AR symptoms	
Stuffy nose	13 (48.1)
Itchy nose and sneeze	14 (51.9)
Main physical evidence	
Paleness and edema in nasal mucosa	6 (22.2)
Redness and edema in nasal mucosa	5 (18.5)
Dry particles	16 (59.3)
Main allergen	
Mite	25 (92.6)
Animal hair	9 (33.3)
Fungi	13 (48.1)
Pollen in spring	19 (70.4)
Pollen in summer/autumn	21 (77.8)
Food allergy	19 (70.4)
AR treatment	
Antiallergic	27 (100)
SLIT	5 (18.5)
SCIT	13 (48.1)
OMA treatment	12 (44.4)
Combination	8 (29.6)
Duration of AR treatment, month	3.5 ± 1.6 (2 – 6)
Follow-up, year	2.4 ± 1.1 (0.5 – 5)
TD/ADHD	
TD	19 (70.4)
ADHD	2 (7.4)
Both	6 (22.2)
Duration from TD/ADHD diagnosis to allergen examination (year)	3.6 ± 1.9 (0.5 – 9)

Note: All patients had eczema as an infant and perennial allergic rhinitis symptoms but more severer during seasons (moderate to severe level). All underwent treatment with anti-allergy medication and home mite removal. Only 1 patient received surgery (not shown in Table 1). AR = allergic rhinitis; TD = tic disorder, severe (n = 12), chronic moderate (n = 7); ADHD = attention-deficient/hyperactivity disorders; SLIT = sublingual immunotherapy; SCIT = subcutaneous immunotherapy; OMAA omalizumab; Combination: sublingual or subcutaneous immunotherapy and OMA treatment.

**Table 2.** VAS, YGTSS, and SNAP-IV scores before and after treatment of AR.

**Variable**	**N**	**Before**	**After**	**p-value**
VAS	27	0.8 ± 0.2	0.4 ± 0.1	< 0.001
YGTSS	25	6.8 ± 1.4	3.5 ± 0.7	< 0.001
SNAP-IV	8	0.6 ± 0.2	0.4 ± 0.1	< 0.001

VAS = visual analogue scale; YGTSS = Yale comprehensive Tic severity scale; AR = allergic rhinitis.
